# Development of a procedure specific and skill based robotic-assisted surgical training program for residents: Delphi study identifying key steps and required skill levels for teaching the low-anterior resection

**DOI:** 10.1007/s00464-025-12072-x

**Published:** 2025-10-03

**Authors:** Y. F. Yvonne Ananias, M. Marije Zwakman, J. P. M. Maarten Burbach, J. Johan Lange, J. P. E. N. Jean-Pierre Pierie, E. C. J. Esther Consten, GROBOT Collaboration Group, M. Marije Zwakman, J. P. M. Maarten Burbach, J. Johan Lange, J. P. E. N. Jean-Pierre Pierie, E. C. J. Esther Consten, L. P. S. Stassen, J. H. W. de Wilt, R. M. P. H. Crolla, D. Roos, J. G. Bloemen, H. L. van Westreenen, J. Melenhorst, B. Grotenhuis, G. van der Schelling, A. B. Smits, H. E. Lont

**Affiliations:** 1 Department of Surgery, University Medical Center Groningen, Hanzeplein 1, 9713 GZ Groningen, The Netherlands; 2 Department of Surgery, Medical Centre Leeuwarden, Henri Dunantweg 2, 8934 AD Leeuwarden, The Netherlands; 3 Postgraduate School of Medicine, University Medical Center Groningen, Hanzeplein 1, 9713 GZ Groningen, The Netherlands

**Keywords:** Surgical training, Robotic surgery, Colorectal

## Abstract

**Introduction:**

The rapid expansion of robotic-assisted surgery (RAS) necessitates comprehensive training of residents. Historically, training programs focused on teaching essential technical skills, neglecting procedure-specific proctor courses. Establishing a step-by-step framework for procedural training promises a uniform, safe, and efficient teaching process. This study aims to identify the key steps and complexities crucial for teaching the robotic-assisted low-anterior resection (RA-LAR) effectively and thereby enhancing the national standardized RAS training program for surgical residents in the Netherlands.

**Methods:**

A list of operation phases and procedural key steps to perform the RA-LAR was compiled, together with a description of four skill levels. Using the Delphi method, experts rated the procedural key steps on a Likert scale and granted them one of four skill levels. The operation phases were queried through multiple-choice questions. Round one, two, and four consisted of online questionnaires; round three, an online meeting.

**Results:**

Consensus was achieved after four rounds. Of the 21 invited experts, 13 participated in the first round (62%) and 11 in the second round (52%). From these 11 experts, 9 (82%) completed the third and fourth rounds (100%). High internal consistency among experts was indicated in Delphi round one by Cronbach’s alpha values of 0.94 for procedural key steps and 0.86 for skill levels. 11 operation phases, 27 procedural key steps, and corresponding skill levels were established for the RA-LAR.

**Conclusion:**

For the RA-LAR, national consensus was reached on the operation phases, procedural key steps, and their corresponding skill levels. A teaching framework is now ready for testing efficacy in training of surgical residents in robotic-assisted surgery.

**Supplementary Information:**

The online version contains supplementary material available at 10.1007/s00464-025-12072-x.

## Introduction

With the rapid expansion of robotic-assisted surgery (RAS) a necessity arises for the comprehensive training of residents [1]. In response to this exigency, the Netherlands is currently in the process of developing a novel RAS training program. The overarching objective of this endeavor is to establish a uniform and standardized RAS training program that seamlessly integrates in current resident training programs and facilitates general as well as procedure-based training across various medical disciplines such as general surgery, urology, and gynecology.

Throughout the years, multiple training programs have been devised for RAS. These mainly feature elements of e-learning, dry- and wet-lab exercises, and virtual reality simulation [2–12]. These programs primarily focus on teaching essential technical knowledge and skills such as hardware and software manipulation, emergency protocol execution, and training manual dexterity by (virtual) tissue manipulation and suturing. Remarkably, scant literature has been disseminated regarding procedure-specific RAS training programs that involve patients. For this purpose, industry offers procedure-specific proctor courses to experienced robotic surgeons, but these are minimally described in literature and are typically inaccessible to those with limited or no prior robotic experience such as residents.

A step-by-step procedure-specific training framework potentially offers a safe, efficient, and uniform learning process for those with little experience. This approach aligns with the principles of cognitive learning theory in which division of a comprehensive body of knowledge into discrete, sequential steps allows for optimal processing and retention within the working memory [13]. Implementation of a standardized training program featuring structured procedural modules further potentially allows to track progress and mitigates the risk of communication errors between surgeon and resident, thereby enhancing the overall efficacy and safety of the training process.

Building a procedure-specific training program starts with identifying key steps of a task (operation) at hand. Preferably based on expert consensus. Already, some work has been done to identify key steps for teaching for the robotic-assisted minimally invasive esophagectomy and robotic-assisted radical prostatectomy [14, 15]. However, another high-volume and educational procedure such as the low-anterior resection (LAR) has only been analyzed in consensus studies focusing on surgical quality [16]. This lacks providing a structured, stepwise, complexity-based training format to safely teach less experienced trainees.

A complexity-based stepwise teaching framework aims to safely extend tasks for trainees by recording and visualizing their skill development and progression through a, preferably procedure-specific surgical curriculum. Although literature informs on what surgical training programs should contain, these are often not procedure specific and emphasize the lack in competence-guided classification systems to assess surgical skills in all stages of training [14, 17, 18]. This recent systematic review even excluded robotic-assisted procedures, thereby presumably underestimating the need for competency-based training and assessment in robotic surgery since this presents a relatively new situation to both trainees and supervisors.

Therefore, the aim of this study is to attain national expert consensus on identifying teaching key steps of the robotic-assisted low-anterior resection (RA-LAR) and assign skill levels to each in order to build a complexity-based stepwise teaching framework integral to the safe, structured, and efficient procedure-specific training of surgical trainees.

## Material and methods

### The Delphi methodology

The Delphi methodology was employed to identify and establish consensus on operation phases and key steps that can be used pragmatically to teach the RA-LAR to surgical residents in a clinical environment. This technique, characterized by anonymity and discussion and group collaboration in later stages, involves presenting statements or ideas to a panel of experts through a structured questionnaire(s) [19, 20]. Subsequently, experts’ responses and comments are meticulously analyzed and subjected to iterative rounds of acceptance, rejection, or further exploration until a collective consensus is achieved [19, 21]. The Delphi methodology fosters an environment that encourages candid and unbiased responses, free from the influence of external opinions or the hierarchy of expertise [19].

### Expert panel

An expert panel was selected to represent currently practicing surgeons in the field of gastrointestinal robotic-assisted surgery in the Netherlands. All experts have more than five years of experience in teaching residents and have performed more than 50 robotic-assisted colorectal procedures. Current literature has no consensus regarding the requisite number of experts for conducting a Delphi survey. For this study, 21 experts from 16 teaching hospitals in the Netherlands were invited to participate via email. Over the span of 2 months, prompt reminders were dispatched to non-responsive invitees via email. Importantly, panel members remained uninformed of the identities of their fellow participants during round one and two.

### Preparation

To establish a constructive step-by-step teaching framework for the RA-LAR, the complete surgical procedure would first be subdivided into potential operation phases. Subsequently, each phase would further be broken down into potentially relevant individual procedural key steps. A list of operation phases and procedural key steps to perform the RA-LAR was compiled, based on guidelines, surgical textbooks, and Incision Academy (Appendix 1) [22]. Each phase or key step identified from these sources was incorporated in the initial questionnaire. In the final questionnaire, each potential individual step would be assessed in terms of its surgical and teaching complexity by asking experts to assign a skill level.

### Skill levels

For this study, a novel classification system was established to discriminate between levels of required knowledge and skills of trainees (i.e., competence) needed to safely learn, perform, and teach each operation key step. Four consecutive subjective skill levels were defined ranging from “basic” to “low complexity,” “fair complexity,” and “high complexity.” Basic skills encompass fundamental surgical techniques achievable by residents with basic (robotic) training and are safe to start teaching. Highly complex skills involve tasks with substantial risks and/or could potentially lead to adverse outcomes or are those that are difficult to teach to trainees without comprehensive experience (Table 1).

**Table 1:**
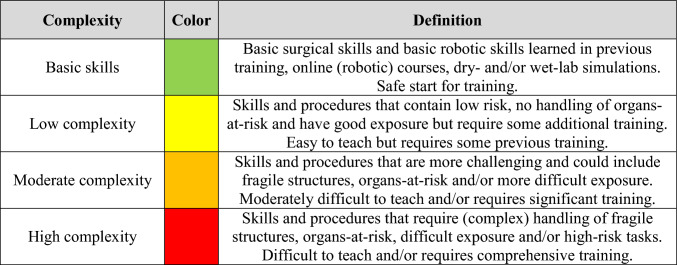
Definitions of skill levels

### Delphi round 1

Questionnaires were distributed via email to experts and reminders were sent. Experts were queried regarding the necessity of including proposed operation phases in the final training program. Optionally, experts could propose additional phases through comments. Regarding the potential key steps, the experts were asked to rate to what extent they believed it was considered relevant to include each as an individual key step in the teaching framework [23]. A Likert scale from one (not important at all) to five (essential) was used. For assessment of skill levels, experts were asked to assign one of four skill levels to each potential key step. To enrich the evaluation process and capture nuanced insights, experts were encouraged to provide supplementary comments whenever deemed necessary. Answers were collected anonymously. After statistical analyses of potential operation phases, key steps, and their assigned skill levels, each was either included, excluded, or required further evaluation in a second questionnaire round. Data and comments received were also considered. New or changed phases or procedural key steps could be added to the questionnaire in round two if deemed necessary by the authors or experts.

### Delphi round 2

The questionnaire in the second round consisted of only potential operation phases and key steps that needed reassessment or those key steps that were already included but did not yet reach consensus on their skill level assignment. Experts were again invited to provide supplementary comments on operation phases and key steps, thereby enriching their Likert scale scores with valuable feedback. As distinguishing between responders and non-responders was not feasible in the context of the anonymous online questionnaire, the decision was made to distribute the second-round questionnaire to all experts uniformly. Additionally, the experts’ contact information was requested to facilitate a potential online meeting with the respondents in the third round. Statistical analysis was carried out conforming to the first round and could again lead to inclusion, exclusion, or need for reassessment of (potential) operation phases, key steps, and skill levels.

### Delphi round 3

In the third round, an online meeting mediated by the research team was arranged to facilitate dialogue and reach consensus between the experts on in- or exclusion of (potential) operation phases, key steps, and skill levels. Two separate online meetings were scheduled with the responders of round two in smaller groups to accommodate all participating experts and enhance discussion. During these meetings, a summary was presented on all items that already reached consensus. Subsequently, all operation phases, key steps, and skill levels that had not attained consensus yet were thoroughly deliberated. In case discrepancy still existed between groups, these items would be re-evaluated in round four with the same experts from the online meeting via a questionnaire.

### Delphi round 4

For final consensus, all discrepant answers from round three were summarized and sent to all participants of the group meetings. We predetermined that the Delphi process would conclude after four rounds, regardless of whether consensus was achieved, due to the anticipation that identification of essential operation phases, procedural key steps, and new insights in skill levels would not likely occur in subsequent rounds.

### Data analysis and consensus

All statistical analyses were conducted using IBM SPSS Statistics 29. Descriptive statistics were calculated for operation phases and skill levels, while means, standard deviations, and 95% confidence intervals were determined for the procedural key steps. Internal consistency among experts for the procedural key steps and skill levels of Delphi round one was assessed using Cronbach’s alpha. Operation phases were included in the RAS training program if the affirmative response was ≥ 80%, while phases receiving < 35% were excluded. In accordance with previous literature using the Delphi methodology, a step was accepted as a key step if the lower confidence limit was ≥ 3.00. A step was excluded if the upper confidence limit was < 3.50 [24]. Furthermore, if a skill level received ≥ 80% of the ratings, it was included. Conversely, a score < 10% resulted in exclusion from consideration. In cases where a consensus was not reached based on the aforementioned criteria, reassessment occurred during rounds two, three, and four. In Delphi round four, the answer options which the majority selected were implemented.

## Results

### Expert panel

Ultimately, 13 surgeons (62%) agreed to participate by completing the online questionnaire. Of these experts, 11 (52%) completed the second round. From these 11 experts, 9 (82%) completed the third and these 9 completed the fourth round as well (100%). As the Delphi process was an anonymous process in the first two rounds, only the participants who sent an additional response email and/or participated in the group discussion are known (12 participants). This group consisted of 5 women and 7 men from 10 different teaching hospitals in the Netherlands.

### Delphi round 1

The initial questionnaire contained 10 operation phases and 31 procedural key steps with matching questions about the skill level to be assessed. Consensus in round one was reached on 8 operation phases, 25 procedural key steps, and 5 skill levels. The remaining 2 operation phases, 6 procedural key steps, and 26 skill levels were provided in round 2 for reassessment. One new operation phase was added after assessment of the qualitative feedback provided during the first round. Furthermore, 3 new procedural key steps and 3 new skill levels were suggested for assessment in round two. Two procedural key steps were rephrased (Table 2).

**Table 2:**
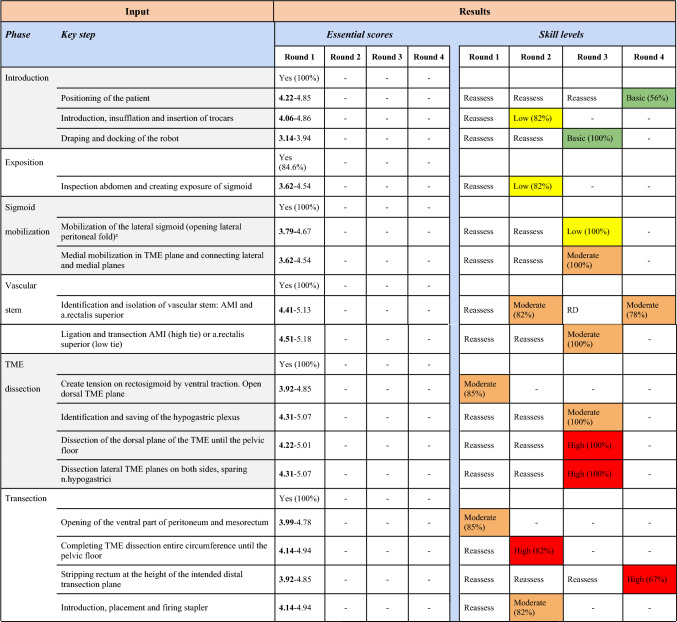

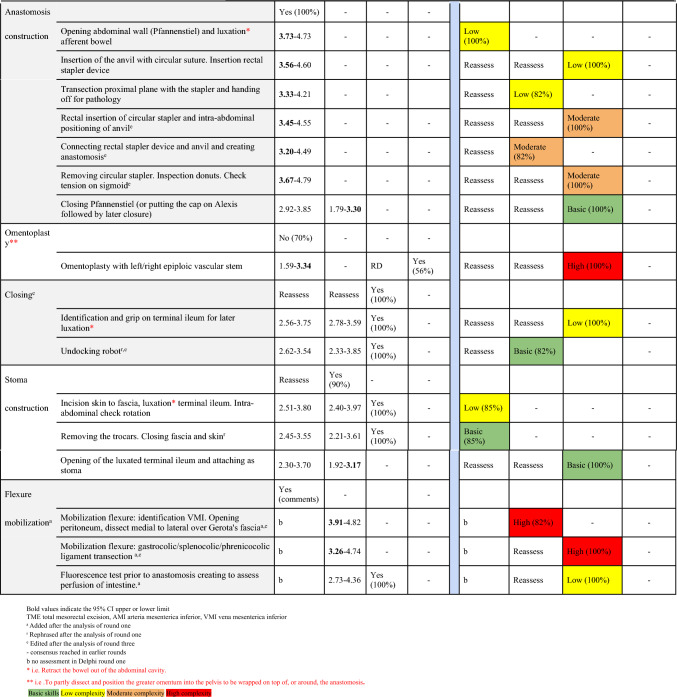
Overview of all four Delphi rounds: operation phases, procedural key steps, and their skill level identified for the RA-LAR

Cronbach’s alpha from Delphi round one was 0.94 for the procedural key steps and 0.86 for the skill levels for the RA-LAR, showing high internal consistency between the experts.

### Delphi round 2

In the second Delphi round, 2 operation phases, 9 procedural key steps, and 29 skill levels needed to be reassessed. One operation phase, 2 procedural key steps, and 10 skill levels were included afterward (Table 2).

### Delphi round 3

In the third Delphi round, one operation phase, 5 procedural key steps, and 19 skill levels needed to be reassessed. One operation phase (after rephrasing), 5 procedural key steps, and 17 skill levels could be accepted afterwards (Table 2). 10 key steps were combined or rephrased after discussion. From previous rounds, 1 accepted skill level and 1 excluded key step caused discussion in round three.

### Delphi round 4

In the fourth round, two skill levels that did not reach consensus in the previous round were reassessed, together with the key step and skill level that caused re-discussion in round three. Consensus was reached on the three skill levels, and the key step was accepted.

## Discussion

This study presents a comprehensive framework for expectedly safe and structured teaching of the robotic-assisted low-anterior resection (RA-LAR). It represents the consensus of experts in minimally invasive surgery and is presented in a clear, stepwise approach with visual tracking of progress for both residents and supervisors. The framework was developed through a four-round Delphi consensus process involving 13 experts from 10 teaching hospitals across the Netherlands. It led to the identification of 11 consecutive operation phases consisting of 27 procedural key steps, each assigned one of four skill levels. This RA-LAR teaching framework is the first of its kind and is designed to become part of a larger educational program aimed at teaching surgical residents various robotic-assisted operations in a structured manner.

Overall, this study found that experts could easily reach consensus on most operation phases and procedural key steps of the RA-LAR in the first round. As the initial research indicated minimal variation in the procedural steps, their inclusion in the analysis was anticipated to contribute limited novel insight. This is likely because the low-anterior resection is a commonly performed procedure laparoscopically and has been previously described in a surgical quality assessment study [16]. Although laparoscopic- and robotic-assisted surgery are both minimally invasive techniques, some differences were identified. In this study, adequate preparation, such as patient positioning and trocar placement, was deemed more important than in laparoscopy. This finding contrasts with a previous laparoscopic study by Dijkstra et al., which suggested a greater tendency to exclude practical and ergonomic steps [24]. Similarly, in the study by Bethlehem et al., preoperative preparation and patient positioning steps generated discussion [25]. This could indicate that in laparoscopic surgery, preparation steps, such as patient positioning, primarily depend on surgical preference and may not significantly influence the performance or quality of the procedures as they do in robotic-assisted surgery.

However, some of the operation phases and procedural key steps required additional rounds to either include, reframe, or remove. Consistent with the literature on laparoscopic procedures, this study found that less commonly performed procedural key steps and corresponding phases, such as creating a stoma or performing an omentoplasty, required multiple rounds to reach consensus [24, 25]. The expert panel was divided on whether to include or exclude these steps as routine in the training, given that they are not part of all RA-LAR operations. Experts in favor of inclusion argued that a description of the required skill level is necessary to avoid potential errors during teaching.

Reaching consensus on assigning skill levels to each of the included key steps was a major point of discussion and sometimes required all four Delphi rounds. Extensive debate highlighted the influence of patient-dependent characteristics, the risk of serious complications, and differences in the confidence and experience of the supervisor. Ultimately, most key steps reached unanimous agreement among experts; however, some did not and were therefore submitted to a final questionnaire in the fourth round. This underscores the challenge of assigning a one-size-fits-all skill level, emphasizing the importance of case-dependent considerations. Consensus was reached on the skill level that would be most appropriate for the majority of cases, providing a guideline for the skill level required for residents to perform these steps safely. Nevertheless, supervisors must remain vigilant to specific patient characteristics and draw on their own teaching experience when coaching residents.

Surgical quality studies often lack tools to visualize the subtle progression of trainees, as each item is evaluated in a binary manner rather than using more detailed metrics that can track performance within each step. Therefore, we designed the skill level system to identify varying complexities between individual key steps, facilitating the development of a stepwise, efficient, and visual framework for teaching environments. Consequently, this study focuses on evaluating the challenges of teaching specific procedural steps to residents, rather than prioritizing optimal surgical outcomes as seen in studies of surgical quality. However, the outcomes of these studies investigating surgical performance are crucial to developing our skill level framework. They provide the foundation on which supervisors must decide whether a trainee can advance to a higher skill level [26–29]. GEARS, a performance score for robotic surgery, evaluates technical skills such as instrument handling, efficiency, and control. It effectively distinguishes between experience levels but includes only one item related to the independence or safety of performance [26]. Laparoscopic tools such as OSATS, GOALS, and PBA, which assess trainee independence, could also be applied to robotic surgery [27–29]. Among these, the PBA score is the simplest and most practical for effectively differentiating skill levels and assessing the safety of each step [30]. Combining GEARS for the evaluation of technical skills and PBA for independence in each step could provide a semi-objective measure to determine trainees’ readiness to advance.

This study has certain strengths and limitations. To our knowledge, this is the first Delphi study reporting on the operation phases and procedural key steps of robotic-assisted low-anterior resection that includes a skill level system designed for teaching purposes. It demonstrates the effectiveness of the Delphi methodology in developing a stepwise framework for robotic-assisted surgery. Anonymized rounds, candid discussions, and group collaboration were used to reach consensus. Compared to other Delphi studies, this study required more rounds to reach consensus, although the number of rounds in Delphi studies reflects only variability in opinion rather than its quality or success [15, 16]. The participating experts represented a sample from 10 teaching hospitals in the Netherlands. Although only 62% of invited experts ultimately participated in the study, 10 experts remain a sufficient number for an adequate Delphi process. While the number of experts included in this Delphi study aligns with commonly accepted standards in specialized surgical research, the relatively small panel size (*n* = 10) may limit the diversity of perspectives captured. Moreover, as the panel consisted of surgeons practicing in teaching hospitals within a relatively small geographic region that offer accredited surgical training programs and routinely perform robotic colorectal surgery, a certain degree of homogeneity was inevitable. This was, however, intentional since it would best reflect the Dutch training environment for which the program is being developed. Nevertheless, these factors may affect the generalizability of our findings to other (international) training settings. Future studies involving larger and more geographically diverse expert panels are advised in case validation is sought to potentially broaden the applicability of the developed training program.

The high Cronbach’s alpha in the first Delphi round indicated that a preliminary consensus on surgical steps already existed before the study began, which facilitated a deeper exploration of the teaching-related aspects, including the assignment of skill levels. The four-tier skill level system, ranging from basic to major complexity, inherently involves the challenge of reaching a specific surgical quality threshold for each step. It provides optimal distinctiveness between skill levels while remaining practical for daily use.

A limitation of the study is that not all prespecified cut-off values could always be met. Nevertheless, group discussions allowed us to reach consensus in most cases; however, in four instances, the majority-selected answer option defined the final skill level or the inclusion of a step. Additionally, this study does not yet demonstrate its applicability in daily practice.

**Fig. 1:**
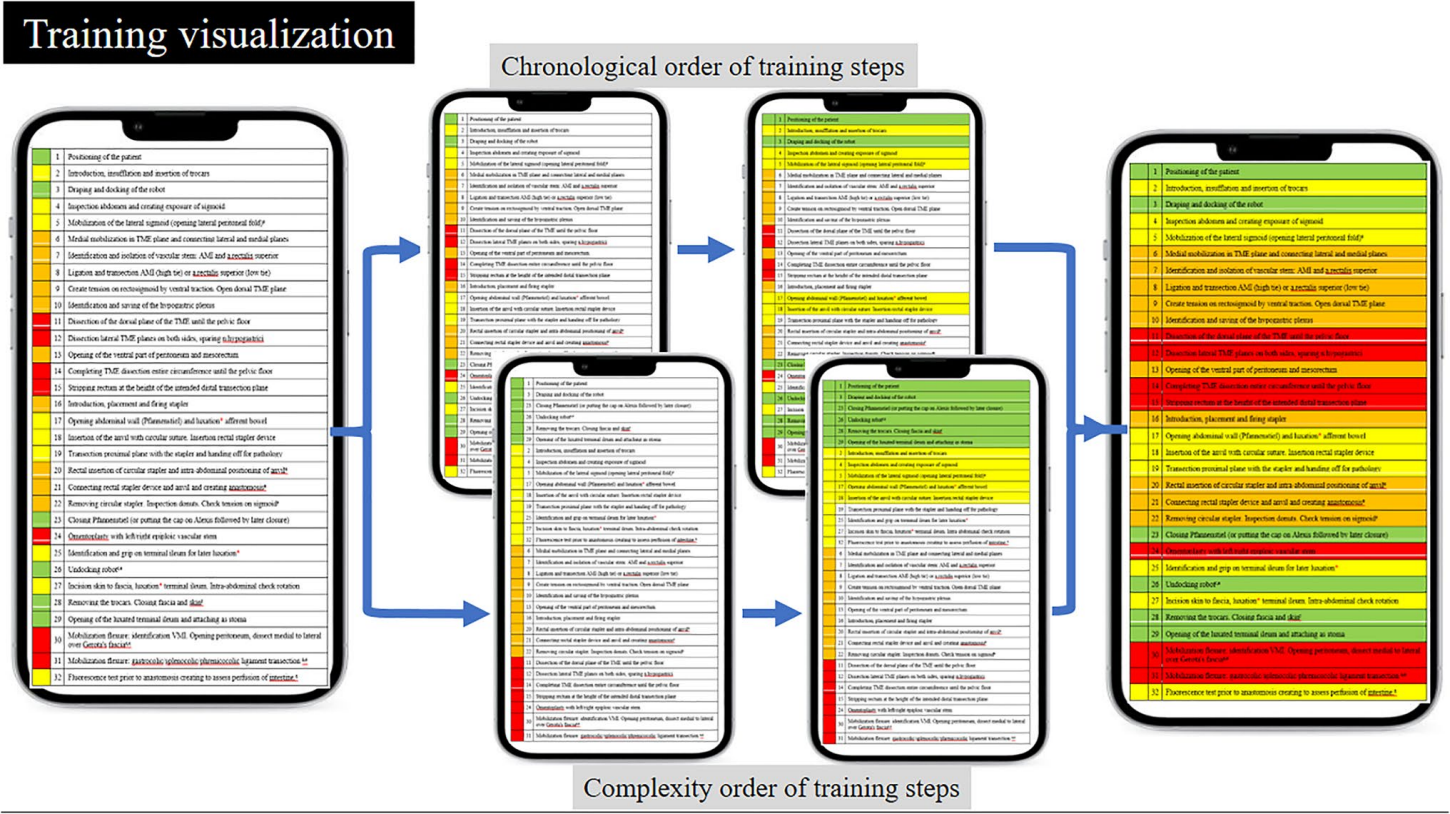
Proposed digital app design of ROBOT-DOCS that enables tracking individual training progression within a procedure over time. Two formats are available to users, either to present steps in chronological order or in complexity (skill level) order. (White background indicates unmastered step, colored background indicates successful completion of step)

This study successfully created a teaching framework for RA-LAR, and further evaluation is now needed to demonstrate its clinical applicability in daily training practice. Through use of the PBA and GEARS scoring systems, the performance of individual steps can be effectively assessed and registered in terms of safety, efficiency, and independence. Trainees register scores for each performed step using the assessment tools, thereby indicating their current skill level. When on a certain skill level, all steps have been marked complete—i.e., the trainees can perform them independently, safely, and efficiently—trainees can progress to a more complex skill level. A visual presentation of our training program in the form of a ‘performance ladder’ that can be ‘climbed’ by trainees (as presented in Fig. 1) will help to evaluate residents’ progress over time in real time. When used, the application will enable trainees to identify their personal next logical learning goal for each new upcoming case. For researchers, it will provide real-time data to monitor app usage, trainee participation rates, and the progression of trainees through the steps in daily practice. The framework or assessment tools do not make a distinction between being competent (i.e., to be able to safely perform a step) and mastery (i.e., to be able to supervise a step) as the PBA and GEARS score systems are not designed to do so.

In conclusion, this manuscript presents the development of a stepwise, skill-based teaching framework for RA-LAR, detailing the essential operation phases and procedural key steps along with their respective required skill levels. Once its feasibility and clinical applicability in daily practice are established, new Delphi studies will be initiated to investigate other procedures.

Based on these results, the ultimate goal is to continue developing a comprehensive, procedure-specific, and skill-based training program for residents in robotic-assisted surgery. This program, referred to as ROBOT-DOCS (ROBOT Dedicated Operation Curriculum for Surgical Trainees), will be expanded by analyzing and incorporating additional procedures in future.

## Conclusion

Through the Delphi methodology, national consensus was reached on identifying operation phases, procedural key steps, and their corresponding skill levels for the robotic-assisted low-anterior resection to aid teaching of surgical trainees.

## Supplementary Information

Below is the link to the electronic supplementary material.

Supplementary file1 (DOCX 14 KB)

## References

[1] Brinkman W, de Angst I, Schreuder H, Schout B, Draaisma W, Verweij L et al (2017) Current training on the basics of robotic surgery in the Netherlands: time for a multidisciplinary approach? Surg Endosc. 10.1007/s00464-016-4970-2 27194262 10.1007/s00464-016-4970-2 PMC5216079

[2] Intuitive Surgical (2017) Intuitive Surgical Da Vinci training. https://www.intuitivesurgical.com/training

[3] Fundamentals of robotic surgery (2017). https://frsurgery.org/. Cited 2022 Apr 4

[4] Roswell Park Comprehensive Cancer Center (2018) Fundamental skills of robot-assisted surgery. https://www.roswellpark.org/education/professional-training/atlas-program/testing-training/fundamental-skills-robot-assisted

[5] Robotictraining.org (2018) Robotics training network. https://robotictraining.org/

[6] Robotictraining.org (2018) Simulated surgical systems. https://robotictraining.org

[7] Intuitive Surgical (2018) daVinci skills simulator. https://intuitivesurgical.com/products/skills_simulator/

[8] Simbionix (2018) RobotiX mentor. https://simbionix.com/simulators/robotix-mentor

[9] Society of American Gastrointestinal and Endoscopic Surgeons (2018) SAGES robotics - masters series. https://www.sages.org/masters-program/

[10] Moit H, Dwyer A, De Sutter M, Heinzel S, Crawford D (2019) A standardized robotic training curriculum in a general surgery program. JSLS J Soc Laparoendosc Surg. 10.4293/JSLS.2019.00045 10.4293/JSLS.2019.00045 PMC6924504 31892790

[11] Kenney PA, Wszolek MF, Gould JJ, Libertino JA, Moinzadeh A (2009) Face, content, and construct validity of dV-trainer, a novel virtual reality simulator for robotic surgery. Urology. 10.1016/j.urology.2008.12.044 19362352 10.1016/j.urology.2008.12.044

[12] Stockheim J, Perrakis A, Sabel BA, Waschipky R, Croner RS (2022) RoCS: Robotic curriculum for young surgeons. J Robot Surg. 10.1007/s11701-022-01444-3 35810233 10.1007/s11701-022-01444-3 PMC10076401

[13] Cnossen F (2015) Cognitive skills in medicine: an introduction. In: Lanzer P (ed) PanVascular medicine. Heidelberg: Springer

[14] Fuchs HF, Collins JW, Babic B, Ducoin C, Meireles OR, Grimminger PP et al (2022) Robotic-assisted minimally invasive esophagectomy (RAMIE) for esophageal cancer training curriculum-a worldwide Delphi consensus study. Dis Esophagus. 10.1093/dote/doab055 34382061 10.1093/dote/doab055

[15] Mottrie A, Mazzone E, Wiklund P, Graefen M, Collins JW, De Groote R et al (2021) Objective assessment of intraoperative skills for robot-assisted radical prostatectomy (RARP): results from the ERUS scientific and educational working groups metrics initiative. BJU Int. 10.1111/bju.15311 33251703 10.1111/bju.15311 PMC8359192

[16] Tou S, Gómez Ruiz M, Gallagher AG, Matzel KE, Amin S, Bianchi P et al (2020) European expert consensus on a structured approach to training robotic-assisted low anterior resection using performance metrics. Colorectal Dis 22(12):2232 32663361 10.1111/codi.15269 PMC7818231

[17] Tonbul G, Topalli D, Cagiltay NE (2023) A systematic review on classification and assessment of surgical skill levels for simulation-based training programs. Int J Med Inform 177:105121 37290214 10.1016/j.ijmedinf.2023.105121

[18] Dell’Oglio P, Turri F et al (2022) Definition of a structured training curriculum for robot-assisted radical cystectomy with intracorporeal ileal conduit in male patients: a Delphi consensus study led by the ERUS Educational Board. Eur Urol Focus 8(1):160–164 33402314 10.1016/j.euf.2020.12.015 PMC9435953

[19] Graham B, Regehr G, Wright JG (2003) Delphi as a method to establish consensus for diagnostic criteria. J Clin Epidemiol. 10.1016/s0895-4356(03)00211-7 14680664 10.1016/s0895-4356(03)00211-7

[20] Dalkey N (1969) An experimental study of group opinion: The Delphi method. Futures 1(5):408

[21] Palter VN, MacRae HM, Grantcharov TP (2011) Development of an objective evaluation tool to assess technical skill in laparoscopic colorectal surgery: a Delphi methodology. Am J Surg. 10.1016/j.amjsurg.2010.01.031 20832048 10.1016/j.amjsurg.2010.01.031

[22] Incision Academy. https://academy.incision.care/.

[23] Rensis Likert. A technique for the measurement of attitudes. Archives of Psychology. 1932;22(140).

[24] Dijkstra FA, Bosker RJ, Veeger NJ, van Det MJ, Pierie JP (2015) Procedural key steps in laparoscopic colorectal surgery, consensus through Delphi methodology. Surg Endosc 29(9):2620–2627 25480611 10.1007/s00464-014-3979-7

[25] Bethlehem MS, Kramp KH, van Det MJ, ten Cate Hoedemaker HO, Veeger NJ, Pierie JP (2014) Development of a standardized training course for laparoscopic procedures using Delphi methodology. J Surg Educ 71(6):810–816 24913426 10.1016/j.jsurg.2014.04.009

[26] Sánchez R, Rodríguez O, Rosciano J, Vegas L, Bond V, Rojas A, Sanchez-Ismayel A (2016) Robotic surgery training: construct validity of global evaluative assessment of robotic skills (GEARS). J Robot Surg 10(3):227–231 27039189 10.1007/s11701-016-0572-1

[27] Martin JA, Regehr G, Reznick R, MacRae H, Murnaghan J, Hutchison C, Brown M (1997) Objective structured assessment of technical skill (OSATS) for surgical residents. Br J Surg 84(2):273–278 9052454 10.1046/j.1365-2168.1997.02502.x

[28] Vassiliou MC, Feldman LS, Andrew CG, Bergman S, Leffondré K, Stanbridge D, Fried GM (2005) A global assessment tool for evaluation of intraoperative laparoscopic skills. Am J Surg 190(1):107–113. 10.1016/j.amjsurg.2005.04.004 15972181 10.1016/j.amjsurg.2005.04.004

[29] PBA Surgery. https://pbasurgery.com/

[30] van Zwieten TH, Okkema S, Kramp KH, de Jong K, Van Det MJ, Pierie JEN (2022) Procedure-based assessment for laparoscopic cholecystectomy can replace global rating scales. Minim Invasive Ther Allied Technol 31(6):865–871 34699305 10.1080/13645706.2021.1995000

