# Correction: Development of a procedure specific and skill based robotic-assisted surgical training program for residents: Delphi study identifying key steps and required skill levels for teaching the low-anterior resection

**DOI:** 10.1007/s00464-025-12562-y

**Published:** 2026-01-20

**Authors:** Y. F. Yvonne Ananias, M. Marije Zwakman, J. P. M. Maarten Burbach, J. Johan Lange, J. P. E. N. Jean-Pierre Pierie, E. C. J. Esther Consten, GROBOT Collaboration Group, M. Marije Zwakman, J. P. M. Maarten Burbach, J. Johan Lange, J. P. E. N. Jean-Pierre Pierie, E. C. J. Esther Consten, L. P. S. Stassen, J. H. W. de Wilt, R. M. P. H. Crolla, D. Roos, J. G. Bloemen, H. L. van Westreenen, J. Melenhorst, B. Grotenhuis, G. van der Schelling, A. B. Smits, H. E. Lont

**Affiliations:** 1 Department of Surgery, University Medical Center Groningen, Hanzeplein 1, 9713 GZ Groningen, The Netherlands; 2 Department of Surgery, Medical Centre Leeuwarden, Henri Dunantweg 2, 8934 AD Leeuwarden, The Netherlands; 3 Postgraduate School of Medicine, University Medical Center Groningen, Hanzeplein 1, 9713 GZ Groningen, The Netherlands

**Correction to: Surgical Endoscopy (2025) 39:8482-8491** 10.1007/s00464-025-12072-x

The original online version of this article was revised to note that coauthors Y. F. (Yvonne) Ananias and M. (Marije) Zwakman contributed equally to the writing of the article.

The original article has been corrected.

