# Supplementary material for: Development of a procedure specific and skill based robotic-assisted surgical training program for residents: Delphi study identifying key steps and required skill levels for teaching the low-anterior resection
Source: Surg Endosc. 2025 Oct 3;40(2):927–36. doi: 10.1007/s00464-025-12072-x (PMC12880992; doi:10.1007/s00464-025-12072-x)
Supplement: Supplementary file 1 — Supplementary file1 (DOCX 14 KB) [file 464_2025_12072_MOESM1_ESM.docx]

**Supplementary table 1: Overview of the initial list of the operation phases and procedural key steps for the RA-LAR.**

| Operational phase | Keystep |
| --- | --- |
| Introduction |  |
|  | 1.Positioning of the patient |
|  | 2. Introduction, insufflation and insertion of the trocars. |
|  | 3. Draping and docking of the robot. |
| Exposition |  |
|  | 4. Inspection of the abdomen and creating exposure of the sigmoid by means of retraction of the omentum and small intestine out of the surgical field. |
| Mobilization of the sigmoid |  |
|  | 5. Lateral sigmoïd mobilization by means of opening the peritoneum over the white line of Toldt. |
|  | 6. Medial opening of the peritoneum of the mesocolon descendens and sigmoid until the rectum (sigmoid take-off). Creating a meso tunnel dorsally of the colon. |
|  | 7. Identification of the ureter on the left side. |
|  | 8. Connecting the lateral and medial planes to get to the TME plane caudally. |
| Care for the vascular stem |  |
|  | 9. Identification and isolation of the vascular stem: AMI and a.rectalis superior. |
|  | 10. Cut through AMI (high tie) or a.rectalis superior (low tie). |
| TME dissection |  |
|  | 11. Make tension of the rectosigmoid and move ventrally. Open TME plane dorsally. |
|  | 12. Identification and sparing of the hypogastric plexus |
|  | 13. Dissection of the dorsal plane of the TME until the pelvic floor. |
|  | 14. Dissection of the lateral plane of the TME on both sides with saving of the n. hypogastrici. |
| Cutting through the rectum |  |
|  | 15. Opening of the ventral part of the mesorectum. |
|  | 16. Finishing of the dissection about the mesorectal fascia through the entire circumference until the pelvic floor. |
|  | 17. Stripping of the rectum at the height of the intended transection plane. |
|  | 18. Cutting through the rectum with a stapler. |
| Construction of anastomosis |  |
|  | 19. Opening of the abdominal wall in accordance with Pfannenstiel and luxation of the leading loop. |
|  | 20. Introducing the anvil with circular stapler and purse-string suture around the anvil. |
|  | 21. Cutting through the proximal transection plane with the stapler and handing of the device. |
|  | 22. Introducing the circular stapler in the rest part of the rectum and position the anvil intra-abdominally. |
|  | 23. Connect the circular stapler and anvil to make the anastomosis. |
|  | 24. Remove the circular stapler and inspect the donuts and create intra-abdominal tension on the sigmoid. |
|  | 25. Close the Pfannenstiel (or put a cap on Alexis). |
| Omentoplasty |  |
|  | 26. Omentoplasty |
| Closing |  |
|  | 27. Identification of the terminal ileum and pick it up to luxate it as a stomalis. |
|  | 28. Ontdocking |
| Creating a stoma |  |
|  | 29. Mark the place of the stoma on the skin. Make an incision in the skin and fascia, luxate the terminal ileum. Check for torsion intra-abdominally. |
|  | 30. Remove the trocarts. Close fascia and skin. |
|  | 31. Open the luxated terminal ileum lis and attach it as a stoma. |
